# A nomogram for predicting 3-year total weight loss percentage following LSG: insights from visceral adipose tissue inflammatory methylation sites

**DOI:** 10.1186/s12893-025-03073-7

**Published:** 2025-07-28

**Authors:** Zhehong Li, Liang Wang, Zheng Wang, Qiqige Wuyun, Buhe Amin, Dongbo Lian, Guangzhong Xu, Nengwei Zhang, Dezhong Wang

**Affiliations:** 1 Beijing Shijitan Hospital, Surgery Centre of Diabetes Mellitus, Capital Medical University, Beijing, 100038 China; 2 Department of General Surgery, Beijing Shijitan Hospital, Capital Medical University, Beijing, 100038 China

**Keywords:** Inflammatory response methylation sites, Percentage of total weight loss, Laparoscopic sleeve gastrectomy, Nomogram

## Abstract

**Background:**

Obesity is a chronic low-grade inflammatory condition. Laparoscopic sleeve gastrectomy (LSG) is a widely recognized intervention for weight management; however, the percentage of total weight loss (%TWL) achieved varies significantly among patients.

**Objective:**

This study aims to develop a nomogram based on methylation sites associated with the inflammatory (INF) in intraoperative visceral adipose tissue (VAT) to predict %TWL at three years post-LSG.

**Methods:**

Patients undergoing LSG were categorized into two groups based on their%TWL three years post-LSG: satisfactory (%TWL ≥25) and unsatisfactory (%TWL<25). Comparative analyses of 850K methylation microarrays from VAT samples were performed to identify methylation sites associated with INF-related genes. Differentially methylated sites were analyzed using least absolute shrinkage and selection operator, random forest, and support vector machine with recursive feature elimination analyses to identify key predictive methylation sites. A nomogram was subsequently developed using these hub methylation sites. The model's performance was assessed through receiver operating characteristic (ROC) curve analysis with bootstrap resampling, calibration curves, decision curve analysis (DCA), and clinical impact curves (CIC).

****Results**:**

Among 25 patients (11 satisfactory and 14 unsatisfactory), 151 differential INF-related methylation sites were identified. Two hub methylation sites, cg14027957 and cg20666492, were selected as predictors for the nomogram. Internal validation demonstrated excellent predictive performance, with an area under the curve (AUC) of 96.8%. The model also showed strong calibration and clinical utility.

****Conclusion**:**

The nomogram, based on two hub methylation sites, effectively predicts%TWL outcomes three years post-LSG. Its high predictive accuracy and clinical relevance suggest significant potential for guiding personalized treatment strategies in patients undergoing LSG

**Supplementary Information:**

The online version contains supplementary material available at 10.1186/s12893-025-03073-7.

## Introduction

Obesity has become a global epidemic, impacting millions of people worldwide [1–3]. Although nutritional interventions, physical activity programs, and pharmaceutical treatments are available, these strategies often fail to deliver sustainable weight loss and metabolic regulation for many patients [4]. Bariatric surgery has emerged as a highly effective approach to managing obesity by reconstructing gastrointestinal anatomy, facilitating significant weight reduction, alleviating obesity-related comorbidities, and improving overall quality of life [5, 6]. Among bariatric procedures, laparoscopic sleeve gastrectomy (LSG) has gained prominence as the most commonly performed surgery worldwide [7].

The success of bariatric surgery is typically evaluated using the percentage of total weight loss (%TWL), with a %TWL threshold of ≥ 25% considered satisfactory [8]. However, 20–30% of patients experience unsatisfactory weight loss or weight regain within the first few years post-surgery [9]. Accurately predicting %TWL outcomes remains a significant challenge. While traditional clinical indicators are readily accessible, their reliability in predicting long-term postoperative outcomes is limited [10]. Recent evidence suggests that early postoperative weight loss post-LSG correlates with long-term weight outcomes [11]. However, reliance on postoperative data introduces a significant time delay, highlighting the need for precise preoperative indicators to predict %TWL effectively.

Obesity is increasingly recognized as a chronic low-grade inflammation condition, characterized by persistent inflammatory responses (INF) within visceral adipose tissue (VAT) [12]. Initially, this inflammatory state acts as a defense mechanism against nutrient excess and an effort to restore metabolic homeostasis. However, over time, it contributes to tissue and organ dysfunction, exacerbating metabolic disturbances [13]. DNA methylation, an epigenetic mechanism that regulates gene expression, maintains genomic stability, and influences disease susceptibility, has been closely associated with obesity and its associated metabolic disorders [14, 15]. Emerging evidence highlights significant associations between imbalanced DNA methylation patterns in adipose tissue and the development of obesity and type 2 diabetes mellitus (T2DM) [16]. Epigenetic studies suggest that specific DNA methylation sites hold promise as biomarkers for predicting, diagnosing, and characterizing obesity and metabolic syndromes. Furthermore, they may play a critical role in optimizing weight loss treatments [17].

Our previous research demonstrated that methylation sites associated with INFs in intraoperative VAT can effectively predict weight changes one year post-LSG [18]. However, predictive factors for %TWL differ across postoperative time points [19], highlighting the need for distinct models to address medium- and long-term outcomes. Transitioning from short-term (one year) to extended prediction models is essential to improve patient care and outcomes. Despite its potential, research into the epigenetic mechanisms linking bariatric surgery and INFs remains in its early stages. Identifying novel epigenetic markers that accurately predict %TWL post-LSG is a crucial unmet need.

This study aims to develop a predictive model based on INF-related methylation sites identified in intraoperative VAT. The model is designed to predict long-term %TWL post-LSG, enabling early identification of patients at risk for unsatisfactory weight loss outcomes. By leveraging these insights, this approach seeks to advance the precision and personalization of post-bariatric surgery interventions.

## Methods

### Study population

**Fig. 1:**
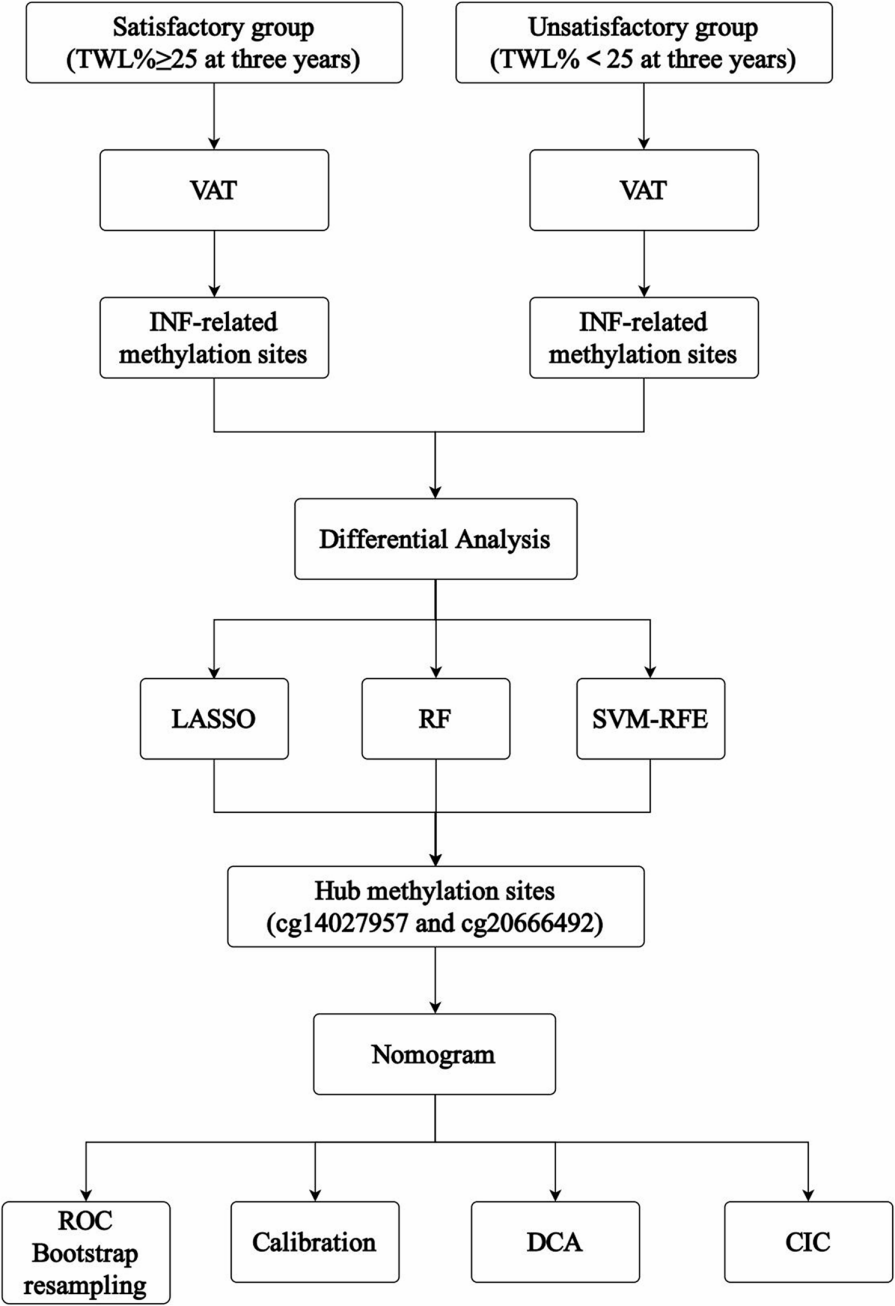
Design and workflow of the study

This study involved a cohort of 25 patients with obesity who underwent LSG at the Centre for Obesity and Metabolic Diseases, Shijitan Hospital. VAT samples were collected from all participants during the procedure. The study protocol was approved by the Institutional Review Board (IRB) of Shijitan Hospital (Approval No. sjtkyll-lx-2019-58) and adhered to the ethical principles outlined in the 1975 Declaration of Helsinki. All study participants have signed an informed consent form, allowing their data and samples to be used for research purposes. An overview of the study workflow is depicted in Fig. 1.

### Clinicopathological characteristics

The inclusion criteria for patients were as follows: (1) body mass index (BMI) ≥ 32.5 kg/m² with or without type 2 diabetes mellitus (T2DM); (2) BMI between 27.5 kg/m² and 32.5 kg/m² with T2DM but failed conservative treatment combined with at least two metabolic diseases or comorbidities; (3) duration of T2DM ≤ 15 years with fasting C-peptide ≥ 50% of the normal lower limit; (4) waist circumference ≥ 90 cm for males and ≥ 85 cm for females; and (5) age between 16 and 65 years. The exclusion criteria included: (1) age < 16 or > 65 years; (2) other types of diabetes (e.g., type 1 diabetes, gestational diabetes, or other specific types); (3) a history of myocardial infarction, cerebral hemorrhage, severe hepatorenal dysfunction, acute infection, or stress conditions in the past three months; (4) pregnant or lactating women; and (5) patients with malignant tumors, a history of liver surgery, serious uncontrollable medical conditions, or mental illness limiting compliance with study requirements.

Supplementary File 1 outlines the LSG procedure. Briefly, pneumoperitoneum (15 mmHg) was established via umbilical Veress needle. Patients were positioned in reverse Trendelenburg with left tilt. Gastric transection began 3 cm above pylorus using 32 Fr Bougie-guided 60 mm staplers, preserving 1 cm at His angle to reduce GERD. No drains were placed. The fascia was closed with absorbable sutures. Following the completion of the LSG procedure, approximately 3 × 3 cm of yellow, VAT was carefully excised from the greater omentum using atraumatic graspers and surgical scissors, with meticulous avoidance of visible vasculature. The management of minor bleeding was accomplished through the utilisation of bipolar electrocautery. The harvested specimen was immediately rinsed in a solution of sterile normal saline. Following this, the specimen was divided into aliquots and flash-frozen in liquid nitrogen for long-term storage at −80 ℃.

Information on the 850 K methylation microarray analysis can be found in Supplementary File 2. Briefly, the DNA of VAT was extracted using a Qiagen Tissue Kit. After assessing purity/concentration (Nanodrop 2000), 500 ng DNA underwent bisulfite conversion (EZ DNA Methylation Gold Kit). Genome-wide methylation was analyzed using Illumina’s MethylationEPIC 850 K array.

Clinical and demographic data collected included sex, age, body mass index (BMI), waist-to-hip ratio (WHR), T2DM, hypertension, hyperuricemia, hyperlipidemia, fatty liver, and marital status. Postoperative follow-up was conducted via telephone interviews. Patients were stratified into two groups based on their %TWL three years post-LSG: the satisfactory group (%TWL ≥ 25%) and the unsatisfactory group (%TWL < 25%).

### Postoperative management and education

Post-LSG T2DM patients were closely monitored to maintain blood glucose within 7.8–10.0 mmol/L, following the 2024 Chinese Clinical Guidelines for Surgery of Obesity and Metabolic Disorders. Follow-up is conducted through online (primarily via WeChat) and offline (outpatient or inpatient) methods, managed by dedicated case managers. Follow-up frequency is adjusted according to time since surgery and patient compliance, with enhanced health education provided for those demonstrating poor adherence.

### Results and significance of methylation sites

The methylation score for each CpG or CpH site was represented as a beta (β) value, calculated using the formula β = (M/(M + U)), where M and U represent the mean methylated and unmethylated signal intensities, respectively. Beta values range from 0 to 1, with “0” indicating no methylation and “1” indicating complete methylation.

### Identification and validation of hub methylation sites

INF-related genes were identified using the gene set enrichment analysis (GSEA) database (available at: http://www.gsea-msigdb.org/gsea/index.jsp, M5932). Methylation sites associated with these genes were extracted from the VAT methylation microarray, followed by differential methylation analysis. To identify key methylation sites predictive of %TWL outcomes (%TWL ≥ 25% or not), we employed three machine learning algorithms: random forest (RF), least absolute shrinkage and selection operator (LASSO) regression, and support vector machine recursive feature elimination (SVM-RFE). LASSO regression, implemented using the “glmnet” package, employed 10-fold cross-validation to determine the optimal penalty parameter (λ). RF, utilizing the “randomForest” package, constructed models with 500 trees and assessed feature importance via Gini impurity. SVM-RFE used 10-fold cross-validation to evaluate feature subsets, selecting the set that maximized cross-validated accuracy. This integrated approach ensures robust identification of key predictive methylation sites.

### Construction and validation of nomogram

A predictive nomogram was developed using the identified hub methylation sites through the “RMS” package in R. Model discrimination was evaluated using receiver operating characteristic (ROC) curve analysis, with bootstrap resampling (1,000 iterations) conducted for internal validation of model performance. Calibration curves were generated to assess the agreement between predicted and observed outcomes. Additionally, decision curve analysis (DCA) and clinical impact curves (CIC) were used to evaluate the clinical utility of the nomogram.

### Statistical analysis

Quantitative variables with normal distributions were compared using t-tests, while non-normally distributed variables were analyzed with the Wilcoxon rank-sum test. Categorical variables were evaluated using either the chi-square test or Fisher’s exact test, as appropriate. All statistical analyses were two-sided, with a p-value of < 0.05 considered statistically significant. Data processing and analysis were performed using R software version 4.2.1.

## Results

### Patient classification and clinical characteristics

The cohort of 25 patients was stratified based on the %TWL three years post-LSG into two groups: (1) the satisfactory group (%TWL ≥ 25%): *N* = 11; and (2) the unsatisfactory group (%TWL < 25%): *N* = 14. No significant differences were found between the two groups in preoperative parameters such as age, sex, WHR, BMI, T2DM, hypertension, hyperlipidemia, hyperuricemia, and fatty liver (Table 1).

**Table 1:** Clinical information of patients in between the satisfactory and unsatisfactory groups

	The satisfactory group (*N* = 11)	The unsatisfactory group (*N* = 14)	*P*
Age (years)	37 (29.5, 44)	31.50 (26.75, 35.5)	0.139
Sex			0.115
Male	5(45.45)	11(78.57)	
Female	6(54.55)	3(21.43)	
WHR	0.89 (0.84, 0.95)	0.99 (0.96, 1.05)	0.006
BMI (kg/m2)	39.49 (38.03, 45.57)	50.32 (47.66, 53.05)	<0.001
Marital status			0.090
Married	1 (9.09)	6 (42.86)	
Unmarried	10 (90.91)	8 (57.14)	
T2DM			1.000
No	3 (27.27)	5 (35.71)	
Yes	8 (72.73)	9 (64.29)	
Hypertension			1.000
No	8(72.73)	10(71.43)	
Yes	3(27.27)	4(28.57)	
Hyperlipidemia			0.208
No	2 (18.18)	7 (50.00)	
Yes	9 (81.82)	7 (50.00)	
Hyperuricemia			0.389
No	5 (45.45)	3 (21.43)	
Yes	6 (54.55)	11 (78.57)	
Fatty Liver			0.695
No	6 (54.55)	6 (42.86)	
Yes	5 (45.45)	8 (57.14)	

### Identification and validation of hub methylation sites

**Fig. 2:**
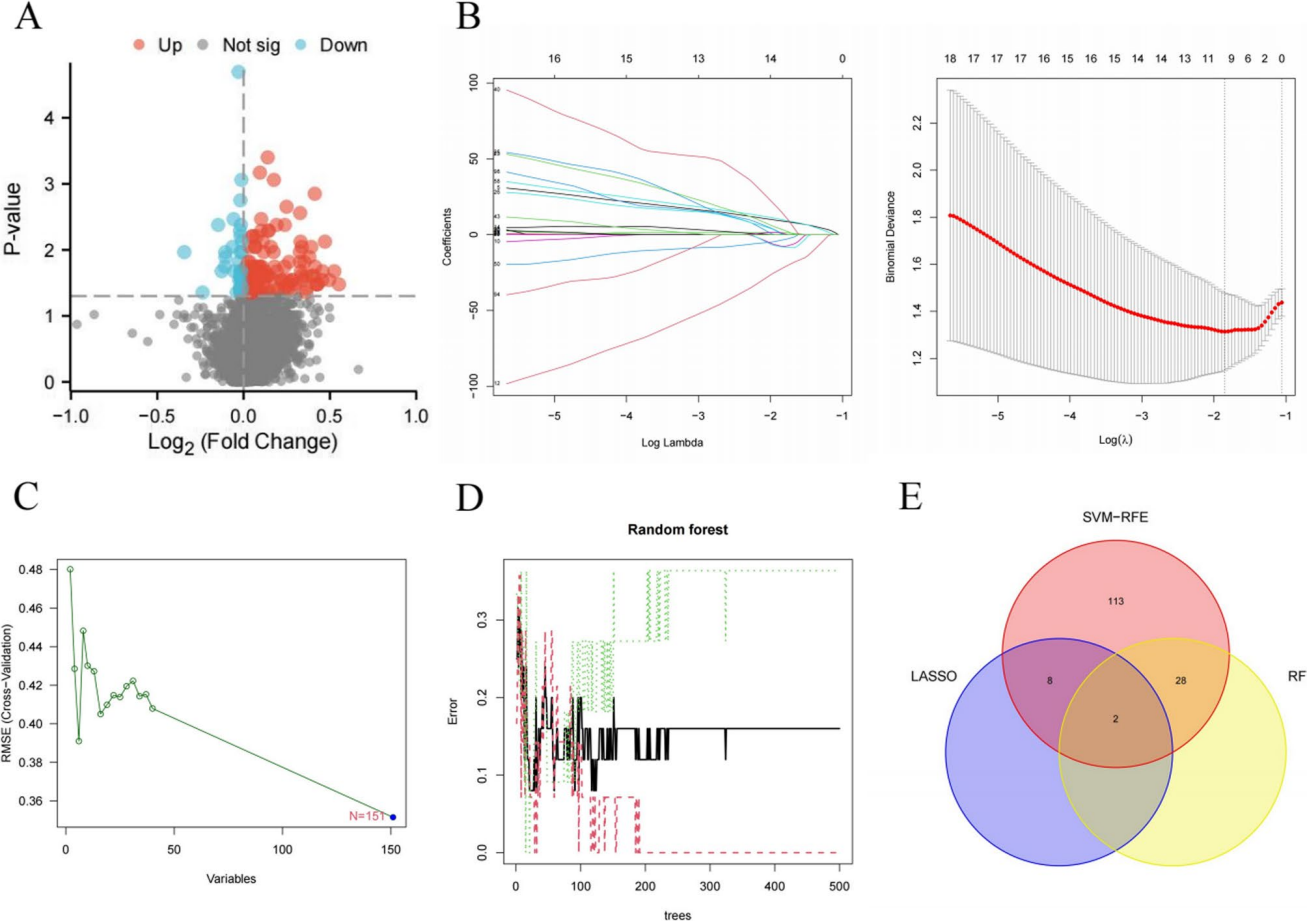
Identification of hub methylation sites **A** volcano plot depicting 151 differentially methylated β-values of INF-related methylation sites between the satisfactory group and unsatisfactory groups. **B **LASSO regression analysis results. **C **SVM-RFE technique algorithm output. **D **RF algorithm output. **E **Venn plot showing the overlap of reliable biomarkers identified by LASSO, SVM-RFE, and RF INF, inflammatory; LASSO, least absolute shrinkage and selection operator; SVM-RFE, support vector machine recursive feature elimination; RF, random forest

**Fig. 3:**
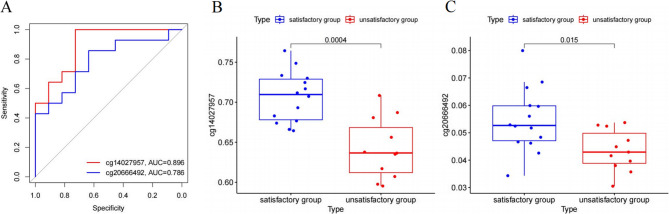
Validation of the Hub Methylation Sites **A **ROC curves assessing the diagnostic ability of cg14027957 and cg20666492. **B **Box plots showing differential expression analysis of cg14027957. **C **Box plots showing differential expression analysis of cg20666492 ROC, receiver operating characteristic

A total of 200 INF-related genes were retrieved from the GSEA database (Supplementary Table 1), leading to the identification of 4,974 INF-related methylation sites. Differential analysis revealed 151 differentially methylated sites based on β-values (Fig. 2A). Key methylation sites were identified using three machine learning algorithms: (1) LASSO regression: 14 methylation sites (Fig. 2B); (2) SVM-RFE: 151 methylation sites (Fig. 2C); and (3) RF: 30 methylation sites (Fig. 2D). Overlap analysis, visualized with a Venn diagram, identified two hub methylation sites: cg14027957 and cg20666492, corresponding to the podoplanin (PDPN) and RAS guanyl-releasing protein 1 (RASGRP1) genes, respectively. The diagnostic utility of these hub methylation sites was assessed using ROC curve analysis. The results showed robust discriminatory performance, with area under the curve (AUC) values of 0.896 for cg14027957 and 0.786 for cg20666492 (Fig. 3A). Additionally, box plots confirmed significant differences in the β-values of these methylation sites between the satisfactory and unsatisfactory groups (Fig. 3B-C, *P* < 0.05).

### Construction and validation of the nomogram

**Fig. 4:**
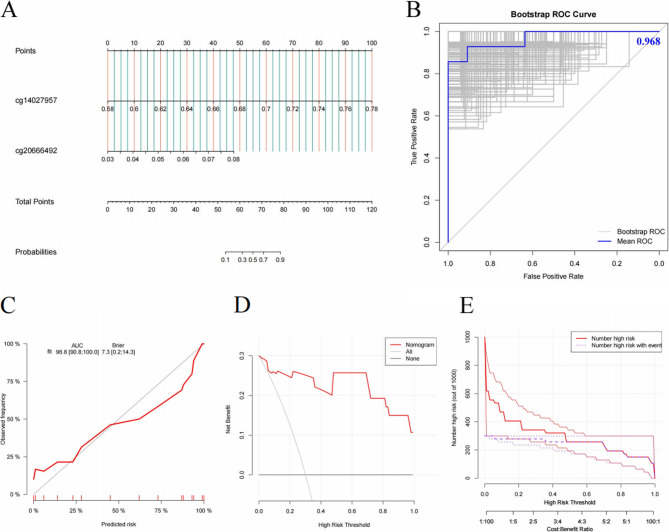
Construction and Validation of the Nomogram **A **Nomogram for predicting long-term outcomes of LSG. **B **ROC curves evaluating the discrimination ability, with internal validation using bootstrap resampling (1,000 iterations). **C **Calibration curve. **D **DCA assessing the clinical utility of the diagnostic model. **E **CIC evaluating the clinical impact of the diagnostic model ROC, receiver operating characteristic; DCA, decision curve analysis; CIC, clinical impact curve

A diagnostic nomogram was developed using logistic regression based on the two identified hub methylation sites (Fig. 4A). Internal validation through 1,000 bootstrap resamplings yielded a mean AUC of 0.968 (95% CI: 0.908–1.000), indicating excellent predictive performance (Fig. 4B). The calibration curve demonstrated strong agreement between predicted probabilities and observed outcomes, with a linear regression slope approaching 1 (Fig. 4C). Additionally, DCA and CIC analysis showed that the nomogram provided a higher net benefit across most threshold probabilities compared to extreme curve scenarios (Fig. 4D-E). This nomogram integrates methylation levels at two CpG sites (cg14027957 and cg20666492) in VAT to predict long-term outcomes after LSG. It assigns scores ranging from 0 to 100 for each methylation site, with their total corresponding to a satisfaction probability between 0.1 and 0.9. Clinicians can utilize this model by (1) obtaining methylation data from adipose tissue samples, (2) plotting individual site scores on the nomogram, (3) calculating the total score, and (4) deriving the predicted satisfaction probability. This tool offers an objective assessment of patient outcomes.

## Discussion

The rapid global rise in obesity has driven a parallel surge in bariatric procedures [20, 21]. However, a 10-year observational study found that 15.7% of patients undergoing LSG required revision surgery [22]. The main reasons for revision include weight regain, insufficient weight loss, and complications such as gastroesophageal reflux, stricture, and persistent metabolic syndrome [23–25]. Therefore, identifying patients at risk of suboptimal weight loss post-LSG is crucial.

Building on our earlier research, which identified hub methylation sites in intraoperative VAT associated with INF that could predict weight changes one year post-LSG [18], we expanded our approach. Recognizing that predictive factors vary across different postoperative time points [19], we employed three machine learning techniques—LASSO, SVM-RFE, and RF—to develop a novel model for predicting long-term %TWL post-LSG.

Obesity and metabolic diseases are frequently characterized by low-grade chronic systemic inflammation [26]. This inflammatory state, triggered by weight gain, shifts the immune system towards a pro-inflammatory response [27]. Adipose tissue, which serves as both an energy storage depot and an immune organ [28], plays a central role in the development of insulin resistance and obesity-related complications through its inflammatory processes [29]. Given the critical involvement of adipose tissue inflammation in the progression of obesity, this study aimed to explore the relationship between INF-related methylation sites and %TWL post-LSG. Based on the previous study design, we matched the preoperative baseline characteristics of patients, and the results showed no statistically significant differences between groups [18]. However, after completing the 3-year follow-up, we observed an intriguing change in baseline characteristics: initial BMI and WHR showed significant differences between groups. This finding not only underscores the importance of BMI and WHR in evaluating postoperative weight changes but also highlights that satisfactory short-term weight loss does not necessarily guarantee sustained long-term outcomes [30]. Furthermore, as the evaluation criteria for metabolic and bariatric surgery outcomes continue to evolve, the assessment framework has shifted from multiple coexisting indicators to a preliminary consensus on using %TWL as a standardized measure, providing a more unified reference for future research [18].

Our investigation identified two central methylation sites—cg14027957 and cg20666492—associated with the genes PDPN and RASGRP1, respectively, which appear to play a role in predicting in the variability of weight loss outcomes following LSG. PDPN is a type I transmembrane sialomucin-like glycoprotein, characterized by a heavily O-glycosylated extracellular domain and a cytoplasmic tail consisting of nine amino acids [31]. Studies have linked PDPN to immune cell infiltration, particularly by neutrophils [32] and macrophag [33]. Recent studies suggest that PDPN + cells in adipose tissue express interleukin-33 (IL-33), a cytokine involved in the immune regulation of T2DM [34–36]. In our study, cg14027957 levels were significantly lower in the satisfactory group compared to the unsatisfactory group, indicating hypomethylation and higher PDPN expression. This suggests a positive correlation between elevated PDPN expression and improved %TWL, making this the first study to establish such a correlation. RASGRP1, previously identified as a genetic locus associated with T2DM risk, is also associated with β-cell dysfunction [37, 38]. Our data revealed that cg20666492 methylation levels were significantly lower in the satisfactory group, indicating a potential epigenetic inhibition of RASGRP1 that may diminished insulin sensitivity and impaired metabolic responsiveness post-surgery. Given the critical role of RASGRP1 in regulating immune and metabolic pathways, its expression may contribute to more favorable weight loss outcomes in selected patients. In conclusion, our findings highlight PDPN and RASGRP1 as potential biomarkers for predicting insufficient weight loss after bariatric surgery. Their methylation status could inform preoperative risk stratification and support the development of individualized treatment strategies aimed at optimizing postoperative success.

Based on these findings, we developed a nomogram incorporating the two hub methylation sites to predict %TWL post-LSG. Given the small sample size, we employed bootstrap resampling (1,000 iterations) for validation, which demonstrated robust predictive accuracy and calibration. The calibration curve showed strong agreement between the predicted and observed outcomes, while DCA and CICs confirmed the clinical utility of the model, highlighting a significantly increased net clinical benefit.

Despite these strengths, our study has limitations: (1) The generalizability of our findings is constrained by the small cohort size and the exclusive inclusion of LSG patients, which precludes comparisons with other bariatric techniques. Larger multi-center studies comparing LSG to alternative procedures are needed; (2) The lack of multiethnic comparative data may restrict the external validity of our results, particularly across different geographic and demographic groups; (3) Technical accessibility: The detection of methylation sites is not yet universally available in clinical settings, and the widespread adoption of this approach depends on future advancements in diagnostic technology.

## Conclusion

Our nomogram, based on cg14027957 and cg20666492, provides a novel tool for predicting long-term weight loss outcomes post-LSG. However, its current dependence on intraoperative VAT biopsies restricts its practical application in the preoperative setting. Despite demonstrating a commendably high AUC, it is essential to recognize the model’s limitations, particularly concerning external validation and applicability across different ethnic groups. This nomogram highlights the critical role of epigenetics in LSG research and underscores the potential for developing personalized treatment strategies.

## Supplementary Information

Supplementary Material 1.

## Data Availability

Data is provided within the manuscript or supplementary information files.
