# Supplementary material for: A nomogram for predicting 3-year total weight loss percentage following LSG: insights from visceral adipose tissue inflammatory methylation sites
Source: BMC Surg. 2025 Jul 28;25:319. doi: 10.1186/s12893-025-03073-7 (PMC12302806; doi:10.1186/s12893-025-03073-7)
Supplement: Supplementary file 1 — Supplementary Material 1. [file 12893_2025_3073_MOESM1_ESM.docx]

**Supplementary file 1. Laparoscopic sleeve gastrectomy procedure**

Pneumoperitoneum (15 mmHg) was created by inserting a Veress needle in the navel. Patients adopted a reverse Trendelenburg and left-side up a position with their legs separated to form an “A” shape. The greater curvature of the stomach is sufficiently dissociated from the His angle by approximately 3 cm above the gastric pylorus. A 32 Fr Bougie tube was placed from the mouth into the stomach. Once the tip of the Bougie tube has passed through the pylorus, the 60 mm endoscopic staplers are placed in position and the gastric antrum is incised with the cartridge attached to the 32 Fr Bougie tube. The stomach tissue was then gradually separated from approximately 3 cm above the pylorus to the His angle, and the entire stomach fundus was removed. Approximately 1 cm of stomach tissue was retained in the His angle to reduce the incidence of gastroesophageal reflux disease. No blue methylene tests or drain tubes were applied. All of the fascial defects were closed with 2-0 absorbable sutures.

**Supplementary file 2. 850 K methylation microarray**

Visceral adipose tissue was collected during the surgery. DNA was isolated from Tissue Kit (Qiagen). The purity and concentration of DNA were estimated using Nanodrop 2000 (ThermoScietific). Approximately 500 ng of genomic DNA from each sample was used for sodium bisulfite conversion using the EZ DNA methylation Gold Kit (Zymo Research, USA), following the manufacturer's standard protocol. According to the manufacturer's instructions, genome-wide DNA methylation was assessed using the Illumina Infinium HumanMethylation850K BeadChip (Illumina Inc, USA). The array data were analyzed using the ChAMP package in R for deriving the methylation level. The methylation status of all the probes was denoted as β-value, which is the ratio of the methylated probe intensity to the overall probe intensity (sum of methylated and unmethylated probe intensities plus constant α, where α = 100).

| Supplementary table 1. Inflammatory response-related genes | | | |
| --- | --- | --- | --- |
| ABCA1 | CYBB | IRF7 | PTGER2 |
| ABI1 | DCBLD2 | ITGA5 | PTGER4 |
| ACVR1B | EBI3 | ITGB3 | PTGIR |
| ACVR2A | EDN1 | ITGB8 | PTPRE |
| ADM | EIF2AK2 | KCNA3 | PVR |
| ADORA2B | EMP3 | KCNJ2 | RAF1 |
| ADRM1 | ADGRE1 | KCNMB2 | RASGRP1 |
| AHR | EREG | KIF1B | RELA |
| APLNR | F3 | KLF6 | RGS1 |
| AQP9 | FFAR2 | LAMP3 | RGS16 |
| ATP2A2 | FPR1 | LCK | RHOG |
| ATP2B1 | FZD5 | LCP2 | RIPK2 |
| ATP2C1 | GABBR1 | LDLR | RNF144B |
| AXL | GCH1 | LIF | ROS1 |
| BDKRB1 | GNA15 | LPAR1 | RTP4 |
| BEST1 | GNAI3 | LTA | SCARF1 |
| BST2 | GP1BA | LY6E | SCN1B |
| BTG2 | GPC3 | LYN | SELE |
| C3AR1 | GPR132 | MARCO | SELL |
| C5AR1 | GPR183 | MEFV | SELENOS |
| CALCRL | HAS2 | MEP1A | SEMA4D |
| CCL17 | HBEGF | MET | SERPINE1 |
| CCL2 | HIF1A | MMP14 | SGMS2 |
| CCL20 | HPN | MSR1 | SLAMF1 |
| CCL22 | HRH1 | MXD1 | SLC11A2 |
| CCL24 | ICAM1 | MYC | SLC1A2 |
| CCL5 | ICAM4 | NAMPT | SLC28A2 |
| CCL7 | ICOSLG | NDP | SLC31A1 |
| CCR7 | IFITM1 | NFKB1 | SLC31A2 |
| CCRL2 | IFNAR1 | NFKBIA | SLC4A4 |
| CD14 | IFNGR2 | NLRP3 | SLC7A1 |
| CD40 | IL10 | NMI | SLC7A2 |
| CD48 | IL10RA | NMUR1 | SPHK1 |
| CD55 | IL12B | NOD2 | SRI |
| CD69 | IL15 | NPFFR2 | STAB1 |
| CD70 | IL15RA | OLR1 | TACR1 |
| CD82 | IL18 | OPRK1 | TACR3 |
| CDKN1A | IL18R1 | OSM | TAPBP |
| CHST2 | IL18RAP | OSMR | TIMP1 |
| CLEC5A | IL1A | P2RX4 | TLR1 |
| CMKLR1 | IL1B | P2RX7 | TLR2 |
| CSF1 | IL1R1 | P2RY2 | TLR3 |
| CSF3 | IL2RB | PCDH7 | TNFAIP6 |
| CSF3R | IL4R | PDE4B | TNFRSF1B |
| CX3CL1 | IL6 | PDPN | TNFRSF9 |
| CXCL10 | IL7R | PIK3R5 | TNFSF10 |
| CXCL11 | CXCL8 | PLAUR | TNFSF15 |
| CXCL6 | INHBA | PROK2 | TNFSF9 |
| CXCL9 | IRAK2 | PSEN1 | TPBG |
| CXCR6 | IRF1 | PTAFR | VIP |
